# Supplementary material for: Adapting CRISPR-associated transposons for rapid and high-throughput reverse genetics
Source: bioRxiv. 2025 Nov 1:2025.10.31.685825. Preprint. [Version 1] doi: 10.1101/2025.10.31.685825 (PMC12636600; doi:10.1101/2025.10.31.685825)
Supplement: Supplement 1 [file media-1.pdf]

a

```
araD  1    ATGTTAGAAGATCTCAAACGCCAGGTATTAGAAGCCAACCTGGCGCTGCCAAAACACAAC  60
sgbE  1    ATGTTAGAGCAACTGAAAGCCGACGTGCTGGCGGCGAATCTGGCGCTTCCCGCTCACCAT  60

araD  61   CTGGTCACGCTCACATGGGGCAACGTCAGCGCCGTTGATCGCGAGCGCGGCGTCT--TTG  118
sgbE  61   CTGGTGACGTTCACCTGGGGCAATGTCAGCGCGGTAGA-CGAAA-CGCGGCAATGGATGG  118

araD  119  TGATCAAACCTTCCGGCGTCGATTACAGCGTCATGACCGCTGACGATATGGTCGTGGTTA  178
sgbE  119  TAATCAAACCTTCCGGCGTCGAGTACGACGTGATGACCGCCGACGATATGGTGGTGGTTG  178
                                                      6    12    18
araD  179  GCATCGAAACCGGTGAAGTGGTTGAAGGTACGAAAAAGCCCTCCTCCGACACGCCAACTC  238
sgbE  179  AGATAGCCAGCGGTAAGGTGGTGGAAGGCAGCAAAAAACCCTCTTCCGATACACCAACGC  238
           30
araD  239  ACCGGCTGCTCTATCAGGCATTCCCCTCCATTGGCGGCATTGTGCATACGCACTCGCGCC  298
sgbE  239  ATCTGGCGCTCTACCGTCGCTATGCCGAAATTGGCGGTATTGTGCATACCCACTCGCGCC  298

araD  299  ACGCCACCATCTGGGCGCAGGCGGGTCAGTCGATTCCAGCAACCGGCACCACCCACGCCG  358
sgbE  299  ACGCCACCATCTGGTCACAGGCCGGGCTGGATCTCCCCGCCTGGGGCACCACCCACGCCG  358

araD  359  ACTATTTCTACGGCACCATTCCCTGCACCCGCAAAATGACCGACGCAGAAATCAACGGCG  418
sgbE  359  ATTATTTTTACGGTGCCATCCCCTGCACGCGACAGATGACCGCAGAGGAGATTAACGGCG  418

araD  419  AATATGAGTGGGAAACCGGTAACGTCATCGTAGAAACCTTTGAAAAACAGGGTATCGATG  478
sgbE  419  AATATGAATATCAGACCGGCGAAGTGATCATTGAAACCTTCGAAGAACGTGGCAGGAGTC  478

araD  479  CAGCGCAAATGCCCGGC-GTTCTGGTCCATTCCCACGGCCCGTTTGCATGGGGCAAAAAT  537
sgbE  479  CGGCACAAAT-CCCGGCGGTGCTGGTGCATTCTCACGGCCCGTTCGCATGGGGTAAAAAC  537

araD  538  GCCGAAGATGCGGTGCATAACGCCATCGTGCTGGAAGAGGTCGCTTATATGGGGATATTC  597
sgbE  538  GCCGCCGATGCCGTGCATAACGCCGTAGTACTCGAAGAATGCGCCTATATGGGTCTATTC  597

araD  598  TGCCGTCAGTTAGCGCCGCAGTTACCGGATATGCAGCAAACG--CTGCTGGATAAACACT  655
sgbE  598  TCGCGCCAGCTTGCGCCGCAGCTCCCTGCGATGCA--AAACGAACTGCTGGATAAGCACT  655

araD  656  ATCTGCGTAAGCATGGCGCGAAGGCATATTACGGGCAGTAA  696
sgbE  656  ACCTGCGTAAGCATGGGGCCAATGCCTATTACGGGCAGTAA  696
```

Identities: 525/701 (75%), Gaps: 10/701 (1%)

b

```
                12    18    24    30
araA CGGAAACCCTGCGTCAGGTCACCCAACATGCCGA
dgoT CCGTAACCCTGCGCCAGGTACCCCACCACCAGCG
```

c

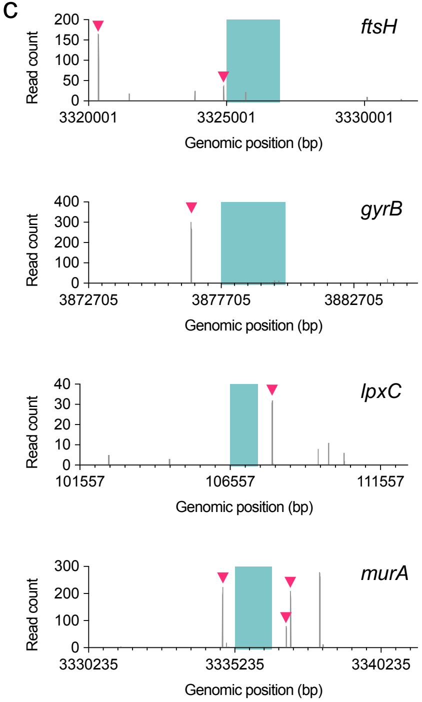

Fig. S1. CAST is susceptible to off-target insertion. a) Sequence alignment between the targeted gene *araD* and the untargeted gene *sgbE*. One guide designed for *araD* shares 25 of 32 nucleotides with a region in *sgbE*, despite lacking a canonical ‘CN’ PAM. The sixth position of the guide is dispensable for target recognition by Type I CRISPR systems. **b)** Sequence comparison between a guide targeting *araA* and its predicted off-target site in *dgoT*. **c)** Off-target insertions can occur when the distance between the guide’s target site and the actual insertion site is unusually long. Such “missed” insertions may inflate estimates of mutant abundance, particularly for essential genes. The coding region of the essential gene is shaded in green. Red arrowheads indicate Read2-mapped insertions associated with Read1 guides targeting the nearby essential gene.

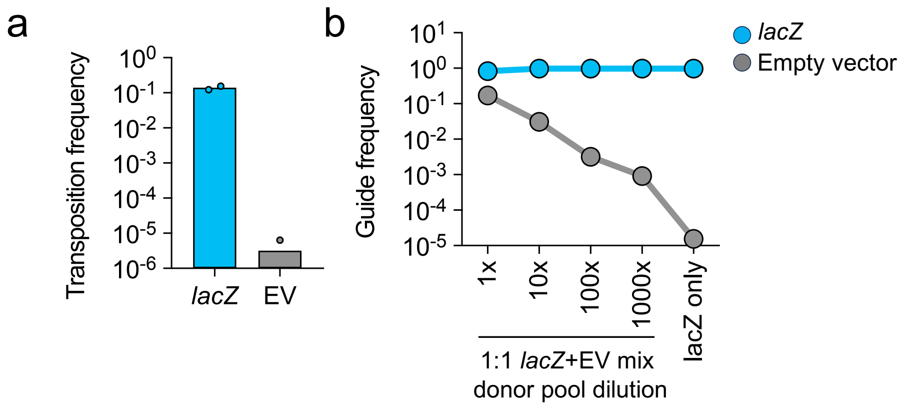

Fig. S2. Independent confirmation of donor plasmid crosstalk. a) Transposition frequency of a non-targeting donor plasmid (empty vector, EV) is >4 orders of magnitude lower than that of a *lacZ*-targeting donor, indicating minimal background activity. **b)** Relative guide frequency of *lacZ*-targeting and EV donor plasmids mixed 1:1 then serially diluted prior to conjugation. Dilution reduces the apparent EV guide frequency measured by PCR, indicating that donor crosstalk overestimates true insertion frequency.

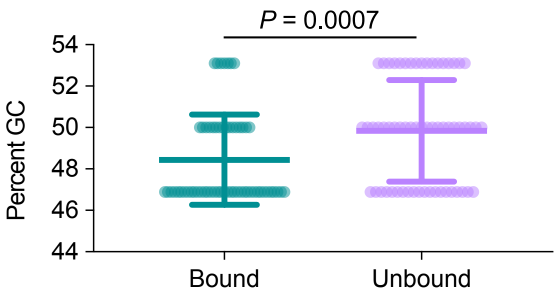

Fig. S3. GC content of guides targeting H-NS bound and unbound regions. Although the difference is statistically different, the average GC content is comparable between guides targeting H-NS-bound regions (48.5%) and those targeting unbound regions (49.8%). Mean ± S.D. is shown. *P* value was calculated using a one-sided Mann-Whitney test.

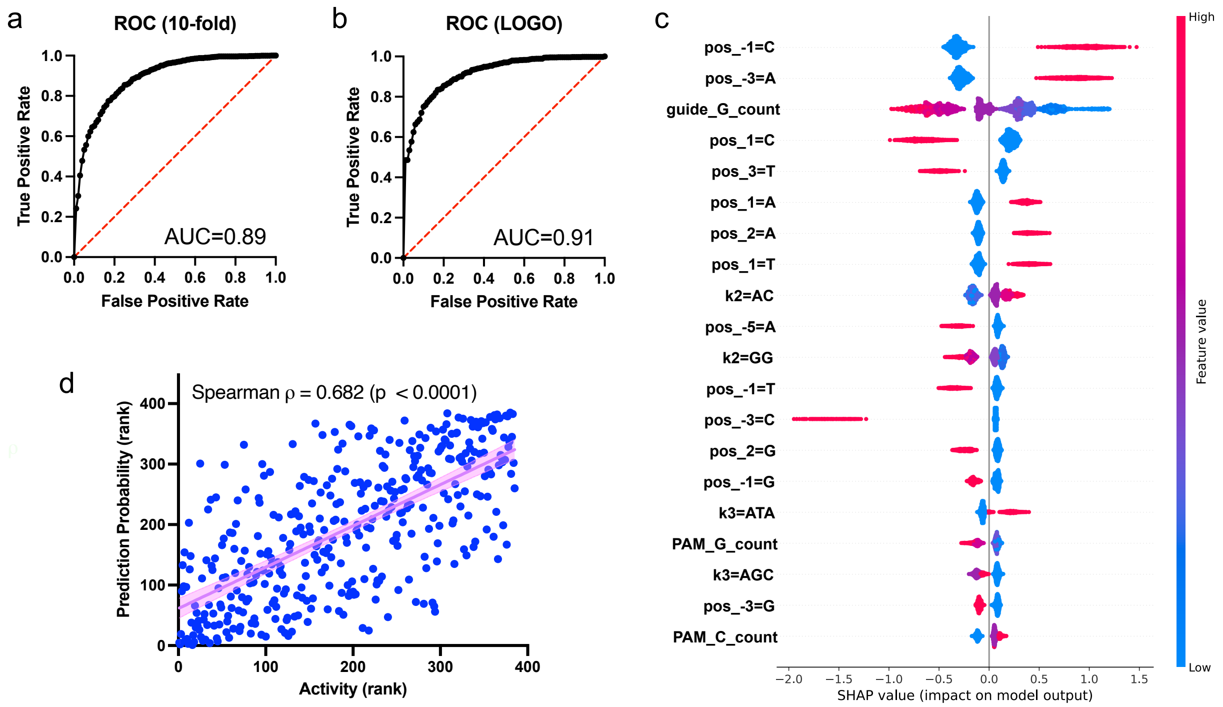

Fig. S4. Internal validation of the predictive model and quantification of feature contributions. Model performance under **a)** 10-fold cross validation and **b)** leave-one-gene-out (LOGO) validation. **c)** Beeswarm plot showing mean SHAP values for the top 20 sequence features most strongly associated with guide activity. **d)** Spearman correlation between observed guide activity and its prediction probability.

Table S1. MultiCAST Pool1 internal validation (10-fold)

fold	n_train	n_test	AUROC	AUPRC	Precision	Recall	F1
1	2685	299	0.8630872	0.8601514	0.7439024	0.8133333	0.7770701
2	2685	299	0.8758837	0.8719454	0.8148148	0.7333333	0.7719298
3	2685	299	0.8887248	0.8748968	0.8297872	0.7852349	0.8068966
4	2685	299	0.8651007	0.8309714	0.7816901	0.7449664	0.7628866
5	2686	298	0.9055448	0.8970441	0.8367347	0.8255034	0.8310811
6	2686	298	0.9050944	0.8860173	0.8175676	0.8120805	0.8148148
7	2686	298	0.8845097	0.8836913	0.7616279	0.8791946	0.8161994
8	2686	298	0.896356	0.8926726	0.8150685	0.7986577	0.8067797
9	2686	298	0.8970767	0.8916902	0.8108108	0.8053691	0.8080808
10	2686	298	0.900545	0.893311	0.775641	0.8120805	0.7934426

Table S2. MultiCAST Pool1 internal validation (leave-one-gene-out (LOGO))

heldout_gene	n_train	n_test	AUROC	AUPRC	Precision	Recall	F1
acrA	2939	45	0.943609	0.826832	0.3181818	1	0.482759
acrB	2930	54	0.945724	0.841906	0.6153846	1	0.761905
ampG	2909	75	0.849673	0.656254	0.5714286	0.83333	0.677966
araA	2920	64	0.951136	0.977636	0.9722222	0.79545	0.875
araB	2905	79	0.912899	0.934305	0.9411765	0.68085	0.790123
araC	2944	40	0.905371	0.868893	0.7142857	0.88235	0.789474
araD	2963	21	0.888889	0.983312	1	0.55556	0.714286
araE	2929	55	0.936937	0.845568	0.6666667	1	0.8
argA	2914	70	0.892992	0.93863	0.9714286	0.70833	0.819277
argB	2955	29	0.847826	0.955394	0.8888889	0.69565	0.780488
argC	2945	39	1	1	1	0.6875	0.814815
argE	2936	48	0.938889	0.958297	0.9565217	0.73333	0.830189
argG	2918	66	0.929101	0.970834	0.9069767	0.86667	0.886364
argH	2923	61	0.962567	0.985389	0.9487179	0.84091	0.891566
cysC	2958	26	0.83125	0.879299	0.8235294	0.875	0.848485
cysD	2955	29	0.789216	0.786605	0.6153846	0.66667	0.64
cysG	2928	56	0.865625	0.770734	0.6315789	0.75	0.685714
cysH	2956	28	0.909091	0.727498	0.5454545	1	0.705882
cysI	2920	64	0.907843	0.916166	0.8181818	0.9	0.857143
cysJ	2913	71	0.909468	0.84855	0.71875	0.82143	0.766667
cysN	2919	65	0.923295	0.920524	0.8709677	0.81818	0.84375
fruA	2924	60	0.919312	0.811181	0.625	0.83333	0.714286
fruB	2946	38	0.955182	0.956479	0.9411765	0.7619	0.842105
fruK	2944	40	0.919271	0.916825	0.625	0.9375	0.75
galK	2936	48	0.927734	0.86362	0.5555556	0.9375	0.697674
galM	2934	50	0.977778	0.751429	0.2777778	1	0.434783
galP	2937	47	0.89819	0.69218	0.5714286	0.92308	0.705882
galR	2949	35	0.894558	0.831984	0.6875	0.78571	0.733333
galT	2937	47	0.87013	0.755159	0.6111111	0.78571	0.6875
hisA	2965	19	0.888889	0.91308	0.8888889	0.8	0.842105
hisB	2939	45	0.942387	0.930813	0.7272727	0.88889	0.8
hisC	2939	45	0.96	0.964388	0.9047619	0.76	0.826087
hisD	2930	54	0.907433	0.878837	0.7894737	0.65217	0.714286
hisF	2955	29	1	1	0.625	1	0.769231
hisG	2953	31	0.850877	0.744847	0.7272727	0.66667	0.695652
hisI	2965	19	0.869048	0.640523	0.6	0.85714	0.705882
ilvA	2936	48	0.96	0.960555	0.95	0.76	0.844444

ilvC	2916	68	0.967777	0.987793	1	0.63265	0.775
ilvD	2913	71	0.901961	0.956137	0.893617	0.82353	0.857143
ilvE	2949	35	0.893939	0.948406	0.8571429	0.75	0.8
lacI	2950	34	0.973333	0.945674	0.6428571	1	0.782609
lacZ	2907	77	0.868673	0.826907	0.6666667	0.88235	0.759494
leuA	2909	75	0.920058	0.949398	0.9705882	0.76744	0.857143
leuB	2947	37	0.773529	0.805406	0.8333333	0.5	0.625
leuC	2932	52	0.93702	0.960322	0.8928571	0.80645	0.847458
leuD	2961	23	0.969231	0.981335	1	0.92308	0.96
metA	2951	33	0.916667	0.932479	0.9047619	0.90476	0.904762
metB	2930	54	0.953846	0.982423	0.969697	0.82051	0.888889
metC	2929	55	0.946779	0.967803	0.8857143	0.91176	0.898551
metE	2902	82	0.897581	0.960921	0.9555556	0.69355	0.803738
metF	2943	41	0.941799	0.97043	0.9545455	0.77778	0.857143
metL	2908	76	0.906154	0.940586	0.8974359	0.7	0.786517
metR	2939	45	0.875	0.947691	0.9545455	0.65625	0.777778
pflA	2962	22	0.717949	0.685744	0.5	0.77778	0.608696
proA	2935	49	0.875	0.794065	0.6363636	0.82353	0.717949
proB	2947	37	0.896825	0.838519	0.5833333	0.77778	0.666667
proC	2959	25	1	1	0.4285714	1	0.6
serA	2929	55	0.978723	0.878292	0.3333333	1	0.5
serB	2952	32	0.94686	0.909453	0.5714286	0.88889	0.695652
thrA	2902	82	0.922101	0.942758	0.8536585	0.76087	0.804598
thrB	2941	43	0.960591	0.983084	1	0.86207	0.925926
thrC	2943	41	0.937799	0.951771	0.8888889	0.72727	0.8

Table S3. MultiCAST Pool3 external validation

AUROC	AUPRC	Precision	Recall	F1
0.871526525	0.99221203	0.9835391	0.7331288	0.8400703

Table S4. Primer sequences for Tn-seq and pooled guide amplification

Tn-seq PCR primers

Name	Sequence
CASTnseq-fwd	AATGATACGGCGACCACCGAGATCTACACTCTTTCCCTACACGACGCTCTTCCGATCTTCATTACT ACTGCAAAGTAGCTGATAAC
P7-AD01-index-Rev	CAAGCAGAAGACGGCATACGAGATCGTGATGTGACTGGAGTTCAGACGTGTG
P7-AD02-index-Rev	CAAGCAGAAGACGGCATACGAGATACATCGGTGACTGGAGTTCAGACGTGTG
P7-AD03-index-Rev	CAAGCAGAAGACGGCATACGAGATGCCTAAGTGACTGGAGTTCAGACGTGTG
P7-AD04-index-Rev	CAAGCAGAAGACGGCATACGAGATTGGTCAGTGACTGGAGTTCAGACGTGTG

Tn-seq oligo adapters

Name	Sequence
Fork truncated-NH2	TACCACGACCA-NH2
Index Fork-rev	GTGACTGGAGTTCAGACGTGTGCTCTTCCGATCTGGTCGTGGTAT

Pooled guides PCR primers

Name	Sequence
Var21	AATGATACGGCGACCACCGAGATCTACACTCTTTCCCTACACGACGCTCTTCCGATCTAATGATG GGTTAAAAAGGATCGATCC
Var22	AATGATACGGCGACCACCGAGATCTACACTCTTTCCCTACACGACGCTCTTCCGATCTATGCGATG GGTTAAAAAGGATCGATCC
Var23	AATGATACGGCGACCACCGAGATCTACACTCTTTCCCTACACGACGCTCTTCCGATCTTGCACGAT GGGTTAAAAAGGATCGATCC
Var24	AATGATACGGCGACCACCGAGATCTACACTCTTTCCCTACACGACGCTCTTCCGATCTTCATTCGA TGGGTTAAAAAGGATCGATCC
Var25	AATGATACGGCGACCACCGAGATCTACACTCTTTCCCTACACGACGCTCTTCCGATCTGAATCGA GATGGGTTAAAAAGGATCGATCC
Var26	AATGATACGGCGACCACCGAGATCTACACTCTTTCCCTACACGACGCTCTTCCGATCTGTCAACTT GATGGGTTAAAAAGGATCGATCC
Var27	AATGATACGGCGACCACCGAGATCTACACTCTTTCCCTACACGACGCTCTTCCGATCTCGGCGTG GCGATGGGTTAAAAAGGATCGATCC
Var28	AATGATACGGCGACCACCGAGATCTACACTCTTTCCCTACACGACGCTCTTCCGATCTCCTGTACC TTGATGGGTTAAAAAGGATCGATCC
AD001	CAAGCAGAAGACGGCATACGAGATCGTGATGTGACTGGAGTTCAGACGTGTGCTCTTCCGATC
AD002	CAAGCAGAAGACGGCATACGAGATACATCGGTGACTGGAGTTCAGACGTGTGCTCTTCCGATC
AD003	CAAGCAGAAGACGGCATACGAGATGCCTAAGTGACTGGAGTTCAGACGTGTGCTCTTCCGATC
AD004	CAAGCAGAAGACGGCATACGAGATTGGTCAGTGACTGGAGTTCAGACGTGTGCTCTTCCGATC

AD005	CAAGCAGAAGACGGCATACGAGATCACTGTGTGACTGGAGTTCAGACGTGTGCTCTTCCGATC
AD006	CAAGCAGAAGACGGCATACGAGATATTGGCGTGACTGGAGTTCAGACGTGTGCTCTTCCGATC
AD007	CAAGCAGAAGACGGCATACGAGATGATCTGGTGACTGGAGTTCAGACGTGTGCTCTTCCGATC
AD008	CAAGCAGAAGACGGCATACGAGATTCAAGTGTGACTGGAGTTCAGACGTGTGCTCTTCCGATC
AD009	CAAGCAGAAGACGGCATACGAGATCTGATCGTGACTGGAGTTCAGACGTGTGCTCTTCCGATC
AD010	CAAGCAGAAGACGGCATACGAGATAAGCTAGTGACTGGAGTTCAGACGTGTGCTCTTCCGATC
AD011	CAAGCAGAAGACGGCATACGAGATGTAGCCGTGACTGGAGTTCAGACGTGTGCTCTTCCGATC
AD012	CAAGCAGAAGACGGCATACGAGATTACAAGGTGACTGGAGTTCAGACGTGTGCTCTTCCGATC
AD013	CAAGCAGAAGACGGCATACGAGATTTGACTGTGACTGGAGTTCAGACGTGTGCTCTTCCGATC
AD014	CAAGCAGAAGACGGCATACGAGATGGAACTGTGACTGGAGTTCAGACGTGTGCTCTTCCGATC
AD015	CAAGCAGAAGACGGCATACGAGATTGACATGTGACTGGAGTTCAGACGTGTGCTCTTCCGATC
AD016	CAAGCAGAAGACGGCATACGAGATGGACGGGTGACTGGAGTTCAGACGTGTGCTCTTCCGATC
AD018	CAAGCAGAAGACGGCATACGAGATGCGGACGTGACTGGAGTTCAGACGTGTGCTCTTCCGATC
AD019	CAAGCAGAAGACGGCATACGAGATTTTCACGTGACTGGAGTTCAGACGTGTGCTCTTCCGATC
AD020	CAAGCAGAAGACGGCATACGAGATGGCCACGTGACTGGAGTTCAGACGTGTGCTCTTCCGATC
AD021	CAAGCAGAAGACGGCATACGAGATCGAAACGTGACTGGAGTTCAGACGTGTGCTCTTCCGATC
AD022	CAAGCAGAAGACGGCATACGAGATCGTACGGTGACTGGAGTTCAGACGTGTGCTCTTCCGATC
AD023	CAAGCAGAAGACGGCATACGAGATCCACTCGTGACTGGAGTTCAGACGTGTGCTCTTCCGATC
AD025	CAAGCAGAAGACGGCATACGAGATATCAGTGTGACTGGAGTTCAGACGTGTGCTCTTCCGATC
AD027	CAAGCAGAAGACGGCATACGAGATAGGAATGTGACTGGAGTTCAGACGTGTGCTCTTCCGATC
